# Systematic analysis of circulating soluble angiogenesis-associated proteins in ICON7 identifies Tie2 as a biomarker of vascular progression on bevacizumab

**DOI:** 10.1038/bjc.2016.194

**Published:** 2016-06-28

**Authors:** Cong Zhou, Andrew Clamp, Alison Backen, Carlo Berzuini, Andrew Renehan, Rosamonde E Banks, Richard Kaplan, Stefan J Scherer, Gunnar B Kristensen, Eric Pujade-Lauraine, Caroline Dive, Gordon C Jayson

**Affiliations:** 1 Institute of Cancer Sciences, University of Manchester, Manchester, UK; 2 Department of Medical Oncology, The Christie NHS Foundation Trust, Manchester, UK; 3 Clinical and Experimental Pharmacology Group, CRUK Manchester Institute, University of Manchester, Manchester, UK; 4 Centre for Biostatistics, University of Manchester, Manchester, UK; 5 Clinical and Biomedical Proteomics Group, St James's University Hospital, Cancer Research UK Clinical Centre, University of Leeds, Leeds, UK; 6 MRC Clinical Trials Unit, University College London, London, UK; 7 Novartis/GSK Pharmaceutical Cooperation, East Hanover, NJ, USA; 8 Department of Gynecological Oncology, Oslo University Hospital, Oslo, Norway; 9 Group d'Investigateurs Nationaux pour l'Etude des Cancers Ovariens (GINECO) and Université Paris Descartes, Assistance Publique–Hôpitaux de Paris, Paris, France

**Keywords:** ovarian cancer, VEGF, bevacizumab, biomarkers, Tie2, Bayesian modelling

## Abstract

**Background:**

There is a critical need for predictive/resistance biomarkers for VEGF inhibitors to optimise their use.

**Methods:**

Blood samples were collected during and following treatment and, where appropriate, upon progression from ovarian cancer patients in ICON7, a randomised phase III trial of carboplatin and paclitaxel with or without bevacizumab. Plasma concentrations of 15 circulating angio-biomarkers were measured using a validated multiplex ELISA, analysed through a novel network analysis and their relevance to the PFS then determined.

**Results:**

Samples (*n*=650) were analysed from 92 patients. Bevacizumab induced correlative relationships between Ang1 and Tie2 plasma concentrations, which reduced after initiation of treatment and remained decreased until progressive disease occurred. A 50% increase from the nadir in the concentration of circulating Tie2 (or the product of circulating Ang1 and Tie2) predicted tumour progression. Combining Tie2 with GCIG-defined Ca125 data yielded a significant improvement in the prediction of progressive disease in patients receiving bevacizumab in comparison with Ca125 alone (74.1% *vs* 47.3%, *P*<1 × 10^−9^).

**Conclusions:**

Tie2 is a vascular progression marker for bevacizumab-treated ovarian cancer patients. Tie2 in combination with Ca125 provides superior information to clinicians on progressive disease in patients with VEGFi-treated ovarian cancers.

Ovarian cancer accounts for 22 000 lives annually in the United States (Jelovac and Armstrong, 2011) and 7000 each year in the UK (Jayson *et al*, 2014). For decades, the combination of surgery and platinum-based cytotoxic chemotherapy has been used to control advanced ovarian cancer (Vergote *et al*, 2010), resulting in response rates of approximately 70% in advanced disease.

As recurrent disease usually occurs within the first 2 years in the majority of patients who present with advanced disease, maintenance or consolidation therapies were tested but were largely unsuccessful. However, randomised trials of anti-angiogenic VEGF pathway inhibitors (Burger *et al*, 2011; Perren *et al*, 2011; Aghajanian *et al*, 2012; Ledermann *et al*, 2013; Pujade-Lauraine *et al*, 2014; Floquet *et al*, 2015; Pignata *et al*, 2015) in ovarian cancer have shown that the addition of such agents to and/or following conventional cytotoxic therapy resulted in improved progression-free survival (PFS) and, in the case of ICON7 (Perren *et al*, 2011; Oza *et al*, 2015), overall survival. On the other hand, VEGF inhibitors can incur moderate toxicity, are expensive and yield somewhat modest improvements in survival. There remains a critical need for biomarkers that predict benefit or progression so that angiogenesis-targeted therapies can be used more effectively.

Ang2 is generated by activated endothelial cells (Gerald *et al*, 2013) and acts as a potential mechanism of acquired resistance to VEGF inhibitors (Daly *et al*, 2013). We reported recently that ovarian cancer patients with supra-median plasma concentrations of Ang1 and infra-median concentrations of Tie2 benefited from bevacizumab (Backen *et al*, 2014), whereas those with raised concentrations of both circulating angiogenesis-associated biomarkers or infra-median concentrations of Ang1 did not benefit. Here, we present the first systematic analysis of angio-biomarker concentrations in the blood of ICON7 patients taken during treatment up to the point that progressive disease was diagnosed. First, we carried out a novel network analysis investigating the correlation of concentrations between angio-biomarkers to discern key changes in angio-biomarker behaviour during treatment. We then applied a hierarchical Bayesian modelling strategy to assess changes in angio-biomarker concentrations to derive a strategy to determine biomarker-based prediction of progressive disease that pertains to patients receiving VEGF inhibitors. The strategy led to a new clinical concept of ‘vascular progression', from which evidence of re-activation of the tumour vasculature can be inferred.

## Methods and statistics

### ICON7 translational research

ICON7 recruited 1528 ovarian cancer patients of whom most (81.5%) had FIGO stage III/IV disease. In ICON7, patients were randomised to receive carboplatin and paclitaxel with or without the anti-angiogenic anti-VEGF antibody bevacizumab, which was administered with and following the cytotoxic therapy. The primary end point was PFS, which was 2 months better in the experimental arm than the control regimen. In a pre-planned analysis, the higher risk patients, who mostly had advanced stage disease, incurred a statistically significant improvement in overall survival of 4.8 months. The response rates for patients receiving the experimental (bevacizumab-containing) or conventional regimen were 67% and 48%, respectively (Perren *et al*, 2011; Oza *et al*, 2015).

Samples for translational research were obtained from all participating Gynaecologic Cancer Intergroup (GCIG) consortia except for the Arbeitsgemeinschaft Gynäkologische Onkologie (AGO). Research projects, utilising ICON7 translational research samples, underwent peer review and were approved by the Trial Management and Steering Committees. The Ethics Committee approved the generic use of translational research samples for angiogenesis and ovarian cancer-related research.

### Longitudinal sample collection and multiplex ELISAs

Blood samples were taken according to differing levels of participation in translational research. Up to 10 samples were taken from each patient. These included two pre-treatment samples and samples taken at the end of the infusion of cycle 1, pre-cycle 2, pre-cycle 6, at the end of cycle 6 infusion, at 6 months, 9 months, 12 months and at progression (see Supplementary Table 1). Plasma (EDTA) was prepared at each centre using standard operating procedures as described previously (Backen *et al*, 2014). Samples were separated into aliquots and frozen at −80 ^o^C, before being shipped to the central sample bank at the University of Leeds, UK where they were stored anonymously at −80 ^o^C. Multiplex ELISAs were used to measure the concentrations of circulating angio-biomarkers including Ang1, Ang2, FGFb, HGF, PDGFbb, VEGF-A, VEGF-C, VEGF-D VEGFR1, VEGFR2, GCSF, IL8, KGF, PLGF and Tie2 using the methods as described previously (Backen *et al*, 2014). Ca125 concentrations were determined at each clinical site.

### Outcome measures

The primary outcome of interest was PFS, defined as the interval from the date of randomisation to the date of disease progression or death, whichever occurred first. Patients who were alive without disease progression at the end of the study were censored at the date of their last assessment. Disease progression was defined clinically or by the RECIST (2010 (http://www.recist.com/index.html); Therasse *et al*, 2000) criteria. Asymptomatic progression on the basis of Ca125 concentrations was insufficient to define progression. The secondary outcome of the trial was tumour response, which was evaluated at cycle 6 of the treatment according to RECIST criteria.

### Statistical analysis

#### Network analysis of angio-biomarkers

Angio-biomarkers are proteins functionally related to the regulation of angiogenesis (Backen *et al*, 2014). The correlations between the concentrations of all angio-biomarkers in response to an angiogenesis-related treatment were determined. Data collected at progression were not included to focus on changes of correlation as a result of treatment effect, while avoiding the biological impact of disease progression and resistance to treatment. We generated network representations of these correlations on the basis of: (i) all patients before treatment, (ii) patients on the standard arm during treatment and (iii) patients on the experimental arm during treatment. Both Pearson's correlation and partial correlation were used to construct the networks, which were plotted using the ‘*qgraph*' package (Epskamp *et al*, 2012) of *R 3.1 (*R Development Core Team, 2014).

#### Illustrating dynamics of angio-biomarkers

The dynamics of each angio-biomarker, measured as log ratios over the baseline, were plotted against the percentage time that elapsed between randomisation and the date of progression/censoring (%PFS; time divided by each patient's PFS interval). Missing data were interpolated for illustration purposes only. At each 10% interval of PFS time, angio-biomarker concentrations in the two arms were compared using Mann–Whitney *U* tests.

#### Correlating angio-biomarker with tumour response

Angio-biomarker concentrations were investigated for their relationship with tumour response, evaluated at cycle 6 of treatment, by comparing angio-biomarkers from patients with complete response with patients with stable disease using Mann–Whitney *U* tests. Patients with partial response were not included to highlight the potential angio-biomarker contrast. The same analysis was carried out for Ca125. Patients with Ca125 concentrations less than 30 IU l^−1^ (tumour undetectable according to GCIG criteria) prior to treatment were not included in these analyses.

#### Bayesian hierarchical modelling of biomarker trajectories

The trajectories of angio-biomarkers were modelled using a Bayesian hierarchical modelling approach from actual rather than interpolated data. It was hypothesised that there was an inflection point in the trajectory of a selected angio-biomarker, which from a clinical point of view was taken to reflect a change in tumour behaviour and therefore as the earliest sign of tumour progression. We approximated the trajectory of an angio-biomarker using a piecewise-linear time relationship where an inflection point separates the decreasing part of the trajectory from the subsequent increasing part. Let *t* denote time measured from treatment start, the piecewise-linear angio-biomarker trajectory for a generic individual was taken to obey:


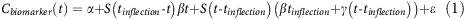


where *C* represents the concentration of a angio-biomarker, *α* is the pre-treatment concentration of the angio-biomarker, *t*_*inflection*_ is the unknown time when inflection occur, *β* and *γ* are the slopes before and after the inflection point. *ɛ* is an Gaussian error term with unknown variance. *S* is an indicator function where


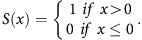


Prior distributions were assigned to these parameters in accordance with the structure of a Bayesian hierarchical model. Any unknown parameters were estimated from the data using a Markov Chain Monte Carlo approach, as implemented in *WinBUGS 1.4 (Imperial College and MRC, UK)*. The rationale of applying this model and details of how the model was implemented can be found in Supplementary Material 1.

#### Developing rules for clinical implementation

On the basis of the Bayesian hierarchical models developed retrospectively using angio-biomarker trajectories, our next step was to develop criteria for using the selected angio-biomarkers in clinic, that is, providing an early prediction of tumour progression while monitoring the angio-biomarkers during treatment. We evaluated rules similar to the GCIG criteria for Ca125, where the elevation of a biomarker with respect to its nadir point is compared with a designated ‘alarm' threshold.

The Bayesian model allowed ‘pseudo-trial' data to be generated via random sampling of the estimated posterior distribution of an angio-biomarker's trajectory (Supplementary Material 1). Instead of creating new pseudo-patients, all the simulated data were generated based on biomarker trajectories of existing patients, as if the trial were repeated on the same patients and data were collected based on new collection scheme. Essentially, this is an inference process similar to interpolating values of missing data points.

Five thousand simulation data were generated per patient per angio-biomarker. Concentrations of angio-biomarkers were imputed on a monthly basis (30±5 days), and were monitored sequentially as if they were measured in clinic. Tumour progression was considered if elevation from the nadir point exceeded a designated cut-off. We sought a threshold value that allowed the angio-biomarker to predict disease progression for as many patients as possible at a time reasonably close to the genuine date of progression, leaving sufficient time for an effective change in treatment to be planned. The performance of different thresholds was compared by plotting the percentage of patients that a biomarker failed to provide any prediction (1–biomarker prediction rate) against time of prediction relative to patient PFS (prediction quality).

## Results

The characteristics of the 92 patients from the ICON7 trial are shown in Table 1. Forty-four patients were in the standard arm, where patients received carboplatin and paclitaxel, and 48 were in the experimental arm, where patients received additional bevacizumab. As expected, the majority of patients had high-grade serous FIGO stage IIIc disease that had undergone cytoreductive surgery leaving less than 1 cm residual disease. Demographic characteristics and histological types for the patients were similar to those of all patients in ICON7 trial. However, the median age of the experimental arm was significantly lower (Kruskal–Wallis test, *P*=0.03). In total, 350 and 300 useable blood samples were provided from patients in the experimental arm (7.3±2.5 samples per patient) and from patients receiving standard cytotoxic therapy (6.8±2.4 samples per patient).

### Ang1-Tie2-VEGF-C regulatory networks induced by bevacizumab

**Figure 1:**
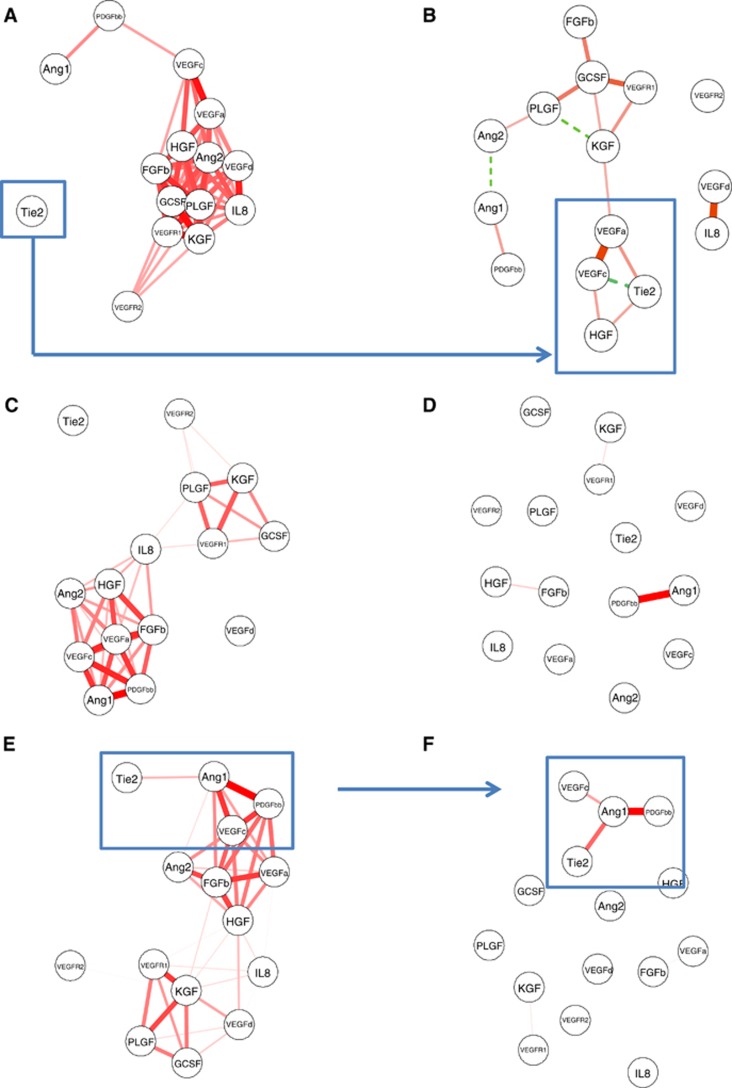
**Analysis of correlation networks.**(**A**) Pearson's correlation network for all patients pre-treatment. (**B**) Partial correlation network for all patients pre-treatment. (**C**) Pearson's correlation network for patients on the standard arm, during treatment. (**D**) Partial correlation network for patients on the standard arm, during treatment. (**E**) Pearson's correlation network for patients on the experimental arm, during treatment. (**F**) Partial correlation network for patients on the experimental arm, during treatment. Pearson's correlation networks were plotted on the left panel (**A**, **C**, **E**) and partial correlation networks were plotted on the right (**B**, **D**, **F**). Each row of plots, from top to bottom, demonstrated the correlation networks for patients at baseline, on standard arm during treatment and on experimental arm during treatment, respectively. Each node in the networks represents one angio-biomarker. The thickness of the edge between two nodes represents the strength of correlation. Positive correlations are red solid lines and negative correlations, green dashed lines. Correlations with absolute values smaller than 0.3 were not displayed. The thickest line represents a maximum correlation of 0.74 while the median correlation shown in the networks is 0.55. Angio-biomarkers are highlighted if they demonstrate significant changes in correlation.

Correlation network analyses were used to identify changes in the correlations of angio-biomarker concentrations that were induced by cytotoxic therapy alone or through the addition of bevacizumab to the cytotoxic regimen. Before treatment, angio-biomarker concentrations formed a highly correlated cluster, suggesting some coordination of biological functions (Figure 1A). Networks from the standard or the experimental arm alone revealed the same topology. Partial correlation analysis (Figure 1B) indicated that Tie2 concentrations correlated with those of VEGF-A, VEGF-C and HGF. The data also suggested a negative correlation between Ang1 and Ang2.

Following treatment with carboplatin and paclitaxel, the pre-treatment angio-biomarker cluster split into two correlated sub-clusters that were weakly linked by IL-8 (Figure 1C). This is consistent with increased tumour cell death, which may have reduced angiogenesis. Partial correlation analysis also demonstrated that correlations between angio-biomarkers were weakened in general (Figure 1D).

In the experimental arm, in which bevacizumab was added, the correlation network remained largely unchanged compared with that from the standard arm, indicating that patients in the two arms were subject to similar treatment effect, for example, cancer cell death. However, the concentration of Ang1 was found to correlate with Tie2 (Figure 1E), and partial correlation analysis (Figure 1F) further revealed a highly positively correlated cluster that included Ang1, Tie2, VEGF-C and PDGF-BB. The positive correlation between PDGF-BB and Ang1 was present in all networks, perhaps indicating a coordinated role in the regulation of vascular maturity, although potentially the central role of platelets and/or change in platelet concentration cannot be excluded as the explanation. This unbiased approach led us to focus attention on the angiopoietin signalling system.

### Plotting angio-biomarker trajectories

**Figure 2:**
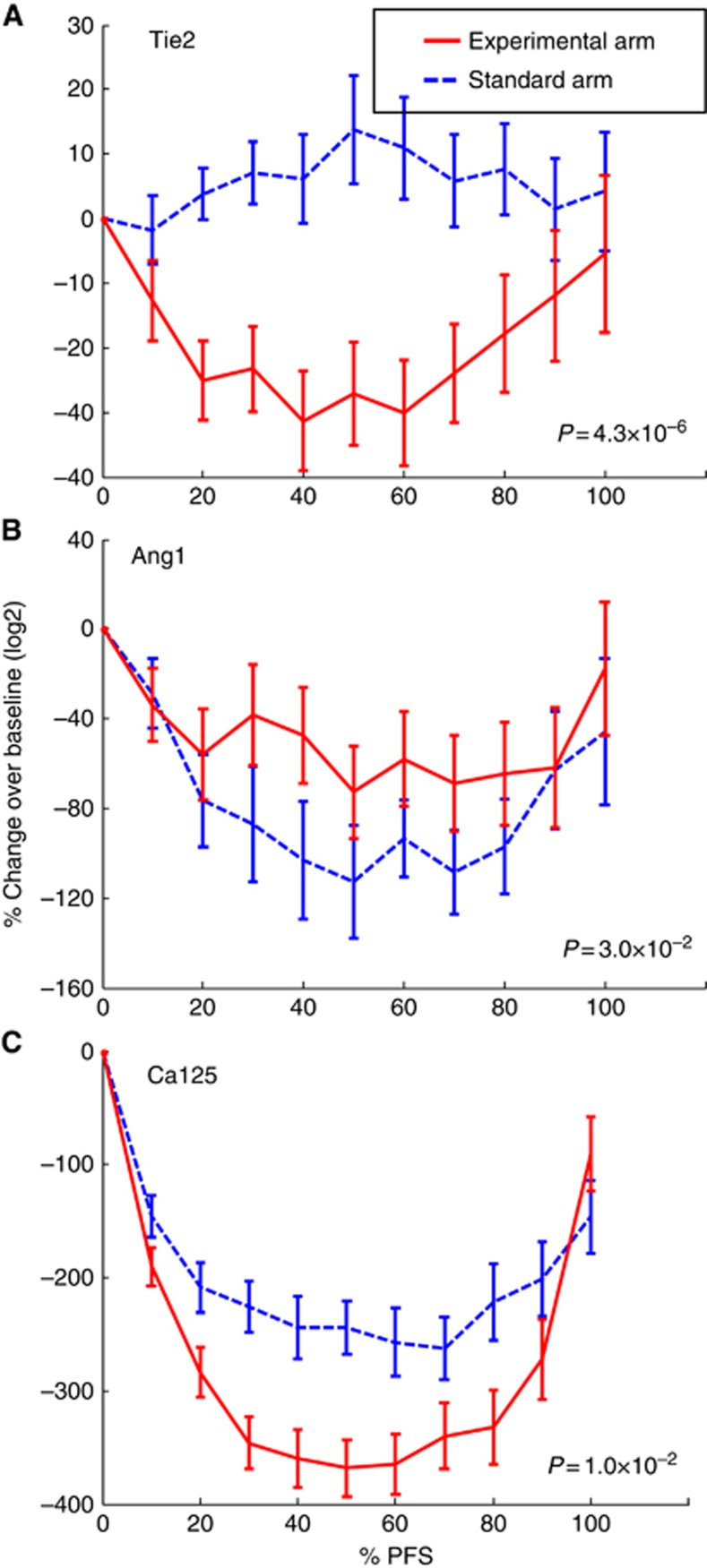
**Mean trajectory of Ang1, Tie2 and Ca125.**The dynamics of Ang1, Tie2 and Ca125, measured as mean percentage change over baseline (log ratio), were plotted against percentage of PFS. The red solid line refers to the experimental arm, and the blue dashed line refers to the standard arm. The data points are presented as ±s.e. Mann–Whitney *U* tests were used to compare the two arms at each 10% time interval and the minimum *P*-value was listed. *P*-values smaller than 0.0003 can be considered as indicating significant difference between the two arms, in accordance with Bonferroni correction for multiple comparison. As Ca125 (2c) was not subject to multiple comparisons, it achieved statistical significance with *P*=0.01. The first time point represents the mean of the two pre-treatment samples.

The concentration–time trajectories of angio-biomarkers are shown in Figure 2 and Supplementary Figure 1. Notably, concentrations of Tie2 reduced in patients treated with bevacizumab, demonstrating a significant difference between the two arms (*P*=7.2 × 10^−6^ at 30% PFS time). However, the difference was no longer significant at the point of disease progression (Figure 2A). Ang1 demonstrated a similar trajectory but the difference between the two arms was not significant (Figure 2B).

As current management of ovarian cancer strongly relies on changes in Ca125 concentrations, we included this ‘gold standard' factor in our analysis. The trajectory of Ca125 concentration during treatment is shown in Figure 2C. Ca125 was more profoundly reduced in the experimental arm (*P*=0.01), presumably because of the superior efficacy of the combined therapy. No significant difference was found in any angio-biomarker between patients with complete tumour response and patients with stable disease.

### Bayesian modelling of biomarker trajectories

We next sought to develop strategies to identify biomarkers that could be used to define progression. This focused our attention on Ang1 and Tie2, which were ligand and receptor, because their concentrations did not correlate until patients were treated with bevacizumab (correlation coefficient of 0.09 pre-treatment and 0.43 post-treatment, respectively). In addition, further evidence to focus on this pathway was attributed to Tie2, which demonstrated the most profound reduction in concentration in bevacizumab-treated patients. These angio-biomarkers were evaluated by applying a Bayesian Markov Chain Monte Carlo modelling approach to investigate the trajectories of (i) Tie2 alone and (ii) Ang1 and Tie2 together, denoted as Ang1 × Tie2, where the two proteins were studied as the product of Tie2 and log2 transformed Ang1 (Supplementary Material 1). Ca125 was also interrogated using the same approach to validate the modelling strategy.

We proposed that an inflection point in the gradient of the concentration–time curve would provide information on the development of progressive disease. Thus, progression for a specific angio-biomarker would be observed when an angio-biomarker concentration that had been decreasing over time, started to increase in concentration. The results of Bayesian modelling confirmed this hypothesis of inflection points for Tie2 and for Ang1 × Tie2 in patients on the experimental arm, but not in patients on the standard arm (Table 2), which is consistent with Figure 2. Thus, bevacizumab has a specific effect on Tie2 and on Ang1 × Tie2, suggesting that Tie2 or Ang1 × Tie2 might be a useful biomarker for vascular activity. On the other hand, Ca125 models were similar for the two different arms, confirming that Ca125 reflects tumour burden, while suggesting that the angiopoietin pathway provides information on vascular biology; a separate compartment.

### Rules for biomarker implementation in clinical practice

**Figure 3:**
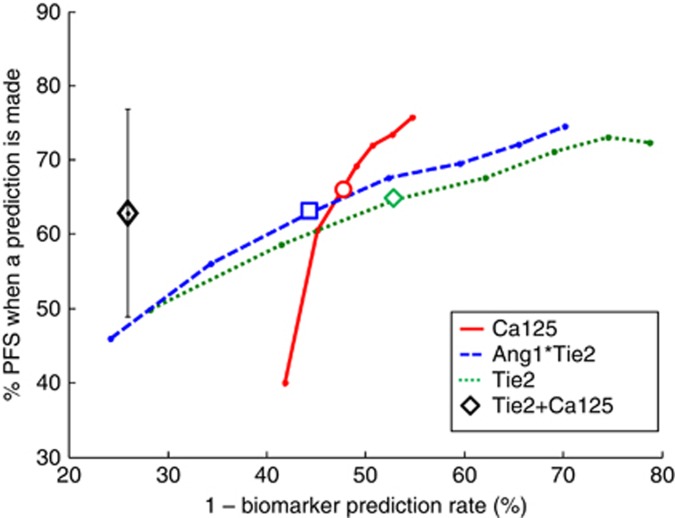
**Tie2 in combination with Ca125 provides better prediction on tumour progression.**The performance of Tie2, Ang1 × Tie2 and Ca125 as biomarkers for predicting tumour progression is shown. 1–biomarker prediction rate is plotted against percentage of PFS time when a prediction is made. Data points close to the top left corner indicate superior performance. The red solid line represents the performance of using Ca125 and the GCIG criteria are shown as a red circle. The green dashed line and the blue dotted line represent the performance of Tie2 and Ang1 × Tie2 as resistance biomarkers, respectively. The highlighted dots on these lines correspond to using 50% elevation from nadir points as criteria for prediction. It demonstrated that if Tie2 was used in conjunction with Ca125, a better prediction can be made compared with using either one alone, as indicated by the black diamond. The combination of Tie2 and Ca125 predicted tumour progression in 74.1% of patients at an average of 62.8%±14.1 %PFS time.

Having developed the methodology for inflection point analysis, we set out to derive rules that were analogous to the GCIG criteria for Ca125 (Rustin *et al*, 2011), allowing us to define angio-biomarker progression. Bayesian models underpinned pseudo-trials for each individual patient in which we developed and tested potential clinical rules for predicting tumour progression. Testing this approach on the validated biomarker, Ca125 (Figure 3), we showed that the optimum behaviour of Ca125 as a biomarker for progression corresponded exactly with the GCIG criteria that define progressive disease (red solid line), increasing confidence in and validating our modelling strategy. The pseudo-trial data predicted progression through analysis of Ca125 to the same extent that we observed in the trial itself (48.9% of patients according to GCIG criteria), also lending credence to our approach.

Our data showed that tumour progression could be predicted in bevacizumab-treated patients if the concentration of Tie2 (or Ang1 × Tie2) increased by 50% from the nadir point during longitudinal measurements (Figure 3), an event we termed ‘vascular progression'. As a biomarker for progression, Ang1 × Tie2 performed only marginally better than Tie2 alone. Therefore, we focused further investigation on Tie2 alone by combining the data with those of Ca125.

By searching for an increase in either Ca125 (GCIG criteria) or Tie2 concentrations (50% increase above nadir) as the criteria for identifying progression, the performance of the progression prediction model improved further, such that progression in 74.1% of patients was predicted, a result that was superior to the performance of either biomarker alone, where less than 50% of progression episodes were predicted (Mann–Whitney *U* test, *P*<1 × 10^−9^). Taken together, our data suggest that simple rules describing Ca125 and Tie2 can be used to identify progressive disease in ovarian cancer patients treated with cytotoxic-bevacizumab combination regimens.

## Discussion

The minimally invasive nature of blood sampling compared with serial biopsy makes routine longitudinal monitoring possible for most cancer patients and such ‘liquid biopsies' are increasingly recognised as pivotal to the development of personalised medicine in oncology. In this study, a correlation network analysis was applied to discern changes in angio-biomarkers that were specific to the effect of bevacizumab. In particular, we showed that Ang1 and Tie2 were co-modulated by bevacizumab with reductions in their plasma concentrations upon the introduction of bevacizumab followed by a return to pre-treatment concentrations as progressive disease occurs.

Our goal was to identify biomarkers that would optimise use of bevacizumab in terms of its efficacy, toxicity and expense. To address this issue, a hierarchical Bayesian Markov Chain Monte Carlo modelling approach was applied to quantify trajectories of angio-biomarker concentrations during treatment. This approach allowed missing data to be handled and was capable of developing optimal rules of applying the angio-biomarkers in clinical implementation. Although successful, greater stability in the data in future studies would be achieved through more frequent, later blood sampling for patients taking part in studies to qualify anti-angiogenic therapy resistance biomarkers.

The data showed that Tie2 or Ang1 × Tie2 are useful predictors of progression in patients receiving bevacizumab but that the small extra benefit of the more complex product of the two angio-biomarker concentrations (Ang1 × Tie2) does not justify its further evaluation. Instead, we were able to develop a simpler biomarker rule, analogous to the GCIG criteria for Ca125 (Rustin *et al*, 2011), in which a 50% increase in plasma Tie2 concentration from nadir was, when used with Ca125 progression criteria, superior to conventional biochemical definitions of disease progression.

This study is based on relatively small cohorts of patients, yet certain findings reinforce the strength of our data: (i) that the changes in Tie2 were restricted to patients treated with bevacizumab and were not seen in those treated with cytotoxic agents implies that the change in Tie2 concentration relates to the effect of bevacizumab on tumour vasculature; (ii) this differential effect was not seen with Ca125, which was modulated in both arms; (iii) such changes in Tie2 have been identified before albeit in the context of the VEGF receptor tyrosine kinase inhibitor, cediranib, when used to treat glioma (Batchelor *et al*, 2010). The latter report is important as the uniform behaviour of Tie2 in different disease settings (ovarian cancer and glioma) and between different VEGF pathway inhibitors (bevacizumab and cediranib) imply that this is a class-relevant finding that pertains to tumour vasculature in general rather than a particular disease type or specific anti-angiogenic treatment.

Our findings demonstrated that the Tie2 changes are seen in bevacizumab-treated patients only and they are not associated with tumour response, suggesting that they are related to tumour vasculature rather than tumour cell compartment. On the other hand, concentrations of Ca125 were associated with the volume of tumour cells. This may explain why a combination of Tie2 and Ca125 provided a superior prediction of tumour progression compared with Ca125 alone. This then identifies a new concept in ovarian cancer, namely that of vascular progression, in which reactivation of the tumour vasculature occurs once the benefit from a VEGF inhibitor has been exhausted. Conceptually this could be important, as we know from recent data that other anti-angiogenic agents are active in ovarian cancer. Our data have identified in the pre-treatment and on-treatment blood samples that the Ang/Tie pathway provides predictive information about the optimum use of bevacizumab. Therefore, we can now ask whether the concept of vascular progression during treatment with VEGF inhibitors identifies the optimum use of Ang/Tie inhibitors in the disease. We know that the most widely evaluated Ang inhibitor, trebananib, is active as a single agent (Herbst *et al*, 2009) in ovarian cancer, yet in phase III trials in biologically unselected patients, the drug was modestly active in one phase III trial (Monk *et al*, 2014). Careful selection of patients based on the biology of their disease will be essential if we are to use anti-angiogenic agents as effectively as possible in ovarian cancer. Thus, the hypothesis to which our work leads is whether the optimum position of Ang/Tie inhibitors in ovarian cancer is when there is evidence of vascular progression based on a rising plasma Tie2 concentration in patients receiving treatment with a VEGF pathway inhibitor. Clearly, further experimental research and prospective clinical trials are now warranted to address this important question and the potential utility of Tie2 as a vascular progression biomarker for bevacizumab in ovarian cancer.

## Figures and Tables

**Table 1:** Patients' characteristics

	**TRICON7**		**ICON7**–**main trial**^a^	
	**Standard (*****N*****=44)**	**Bevacizumab (*****N*****=48)**	**Standard (*****N*****=764)**	**Bevacizumab (*****N*****=764)**
**Age–years**				
Median (range)	60 (38–75)	53 (31–71)^b^	57 (18–81)	57 (24–82)
**Race–*****n*** **(5)**				
White	41 (93)	43 (90)	737 (96)	730 (96)
Asian/Black/Other	3 (7)	5 (10)	27 (4)	34 (4)
**ECOG PS–*****n*** **(%)**^c^				
0	16 (37)	23 (48)	358 (47)	334 (45)
1	27 (63)	23 (48)	354 (47)	366 (49)
2	0	2 (4)	43 (8)	45 (6)
**Origin of cancer–*****n*** **(%)**				
Ovary epithelial	42 (95)	38 (79)	667 (87)	673 (88)
Fallopian tube	1	2	29 (4)	27 (4)
Primary peritoneal	1	8 (17)	56 (7)	50 (6)
Multiple sites	0	0	12 (2)	14 (2)
**Histology–*****n*** **(%)**				
Serous	32 (73)	40 (83)	529 (69)	525 (69)
Mucinous	1	0	15 (2)	19 (2)
Endometrioid	5 (11)	4	57 (7)	60 (8)
Clear cell	5 (11)	3	60 (8)	67 (9)
Mixed	0	0	48 (6)	40 (5)
Other	1	1	55 (7)	53 (7)
**FIGO stage–*****n*** **(%)**				
I/IIA	4 (9)	5 (10)	75 (10)	67 (9)
IIB/IIC	5(11)	2	70 (9)	70 (9)
III	0	1	14 (2)	18 (2)
IIIA	3	1	32 (4)	22 (3)
IIIB	2	8 (17)	44 (6)	45 (6)
IIIC	26 (59)	26 (54)	432 (57)	438 (57)
IV	4 (9)	5 (11)	97 (12)	104 (13)
**Grade–*****n*** (%)				
Grade 1	1	2	56 (7)	41 (5)
Grade 2	9 (20)	5 (10)	142 (19)	175 (23)
Grade 3	34 (77)	41 (85)	556 (74)	538 (71)
Unknown	0	0	10	10
**Debulking surgery–*****n*** (%)				
No (inoperable)^d^	0	0	17 (2)	13 (2)
Yes	44 (100)	48 (100)	747 (98)	751 (98)
>1 cm residual disease	16 (36)	15 (31)	195 (26)	192 (26)
⩽1 cm residual disease	28 (64)	33 (69)	552 (74)	559 (74)
**Intent to start chemotherapy following surgery–*****n*** **(%)**				
⩽4 weeks	13 (30)	23 (48)	328 (43)	326 (43)
>4 weeks	31 (70)	25 (52)	436 (57)	438 (57)
**BMI–kg m**^**−2**^ e				
Median (range)	25.3 (18.4–35.2)	(23.3 17.9–37.6)	Not reported	Not reported
**Smoking**^e^				
Current	2 (5)	4 (8)		
Never/ex-smoker	41 (95)	44 (92)	Not reported	Not reported

Abbreviations: BMI=body mass index; PS=performance status.

a Data from appendix to (Perren *et al*, 2011).

b Kruskal–Wallis test, *P*=0.030.

c Missing data on PS in one patient.

d Inoperable cases were excluded in TRICON7.

e Missing BMI and smoking data in one patient.

**Table 2:** Modelling biomarker trajectories

	***β*** **(slope before inflection point)**			***γ*** **(slope after inflection point)**		
	**Experimental arm**	**Standard arm**	**Mann Whitney** ***U*** **test**	**Experimental arm**	**Standard arm**	**Mann–Whitney** ***U*** **test**
Ang1 × Tie2	−2.9	0.1	**5.3 × 10**^**−4**^	1.2	0.6	**6.0 × 10**^**−7**^
Tie2 only	−3.4	2.0	**5.0 × 10**^**−9**^	0.9	0.6	**2.1 × 10**^**−3**^
Ca125	−4.4	−4.4	0.6	0.9	1.0	0.7

The trajectories of Tie2, Ang1 × Tie2 and Ca125 were modelled using the Bayesian Markov Chain Monte Carlo modelling approach, and the estimated values for major parameters are summarised in the table. In the experimental arm, all putative biomarkers demonstrated negative slopes before the inflection point in trajectories, followed by positive slopes after the inflection point. These results confirmed the validity of the hypothesis on inflection points. In the standard arm, however, significantly different trajectories were observed on Tie2 and Ang1 × Tie2. Only the trajectories of Ca125 were consistent in both arms. The bold values indicate significant *P*-values.
