# Supplementary material for: Systematic analysis of circulating soluble angiogenesis-associated proteins in ICON7 identifies Tie2 as a biomarker of vascular progression on bevacizumab
Source: Br J Cancer. 2016 Jun 28;115(2):228–35. doi: 10.1038/bjc.2016.194 (PMC4947705; doi:10.1038/bjc.2016.194)
Supplement: Supplementary Material 1 [file bjc2016194x1.doc]

**Supplementary material 1**

**Justification of using a Bayesian hierarchical modelling approach**

In this study, we applied a Bayesian hierarchical modelling approach to investigate concentrations of a specific biomarker as a function of treatment time. In principle, a Bayesian modelling approach intended to develop probability models that define joint probability distribution for all observed and unobserved data ^[1]^. This approach has achieved increasing popularity in medical research, and was especially suitable for analysing biomarker data as we present in this study, thanks to its flexibility to produce complicated models with multiple conceptual layers and large numbers of parameters, as well as its natural framework to tolerate missing data points which occur frequently in clinical trials. Similar modelling approach has been applied in recent literature, for modelling trajectories of PSA in prostate cancer ^[2]^ and multivariate clinical factors in Parkinson’s disease ^[3]^.

The Bayesian hierarchical model was utilized to address two questions. Firstly, it provided a framework to define dynamics of biomarker concentrations over time, and in particularly to reflect our hypothesis on inflection point, i.e., there was an inflection point separates the decreasing part of CAB trajectory from the subsequent increasing part. The Bayesian hierarchical model allowed posterior distribution of treatment-time-to-inflection being estimated. Secondly, the model enabled inference of the concentration of a selected CAB at any specific time point for any patient, based on estimated posterior distribution of CAB concentrations. This means that we can carry out pseudo-trials on the same cohort of patients but on different sample collection schemes. It should be aware that this inference process was different from the concept of “prediction”, as it did not generate “new patient data” based on “existing patient data”. Instead such estimation is similar to interpolating values of missing data points. Pseudo-trials can be used to test different clinical rules for biomarkers to predict tumour progression so that optimal practice on optimal selection of biomarkers can be determined.

**Setting up Bayesian hierarchical model and updating it using a MCMC approach**

The Bayesian hierarchical model was setup based on a piecewise linear time relationship, parameterized as listed in *Eq. 1 (*repeated below)


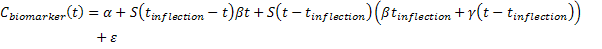


where *C* represents the concentration of the CAB being modelled, *α* is the pre-treatment concentration of CAB that follow a normal distribution; *t_inflection_* is the inflection point of CAB trajectory, which follows a uniform distribution ranging from 30 days after the start of treatment to 30 days prior to disease progression; *β* is the slope before the change point that is dependent on *α* and follows a normal distribution; *γ* is the slope after the change point that follows a normal distribution and *ε* is a random error. *S* is an indicator function where


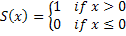


The parameters were assigned the following distributions to follow the structure of a Bayesian hierarchical model:


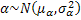


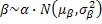


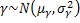


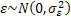


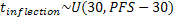


In addition, the *µ* and *σ* of the above normal distribution were assigned appropriate prior distributions in accordance with the CAB being modelled. In this work, these prior distributions were chosen carefully due to limited availability of data points. For example, the following prior values were assigned to Ca125:


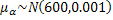


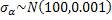


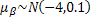


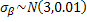


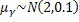


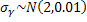


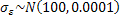


All putative biomarkers being modelled were transformed to check that their dynamics were in the designated range. For example, Ca125 was log2 transformed and then multiplied by 100. Ang1 was also log2 transformed before calculating its product with Tie2.

The posterior distributions of the parameters were determined using an MCMC approach as implemented in Winbugs 1.4. For each model, three Markov chains were trained at a same time. They were updated 50000 times or until model convergence, whichever occurred later. According to our observations, convergence was typically achieved within 10000 update iterations. A converged model was subjected to 30000 further update iterations to estimate the posterior distribution of each parameter. This model allowed us to identify patients with an inflection point, and to compare patients between the two study arms from this point of view.

**Reference**

[1] Gelman A, Carlin JB, Stern HS, and Rubin DB (2004) Bayesian data analysis. Chapman & Hall/CRC.

[2] Zhao L, Feng D, Neelon B and Buyse M (2015) Evaluation of treatment efficacy using a Bayesian mixture piecewise linear model of longitudinal biomarkers. Stat Med, 10; 34(10):1733-46.

[3] Luo S, and Wang J (2014) Bayesian hierarchical model for multiple repeated measures and survival data: an application to Parkinson's disease. Stat Med, 30; 33(24):4279-91.
