# Supplementary material for: Systematic analysis of circulating soluble angiogenesis-associated proteins in ICON7 identifies Tie2 as a biomarker of vascular progression on bevacizumab
Source: Br J Cancer. 2016 Jun 28;115(2):228–35. doi: 10.1038/bjc.2016.194 (PMC4947705; doi:10.1038/bjc.2016.194)
Supplement: Supplementary Table 1 [file bjc2016194x2.doc]

**Supplementary Table 1**

|  | **Collection of plasma level 3** | **Collection of plasma level 4** |
| --- | --- | --- |
| **1+2** | Pre-treatment | Pre-treatment |
| **3** |  | Post cycle 1 (end of infusion) |
| **4** | Pre-cycle 2 | Pre-cycle 2 |
| **5** | Pre-cycle 6 | Pre-cycle 6 |
| **6** |  | Post cycle 6 |
| **7** |  | 6 months |
| **8** |  | 9 months |
| **9** |  | 12 months |
| **10** | Disease progression | Disease progression |

Level 3 and 4 involved provision of samples prior to and during treatment and at progression. In level 4, the most intensive level of blood sample provision, venepuncture occurred twice before treatment to establish intra-patient variability for each CAB, immediately after cycle 6, then at 3 monthly interval for the first year unless or until progressive disease occurred, at which a final blood sample was taken.
