# Supplementary material for: Systematic analysis of circulating soluble angiogenesis-associated proteins in ICON7 identifies Tie2 as a biomarker of vascular progression on bevacizumab
Source: Br J Cancer. 2016 Jun 28;115(2):228–35. doi: 10.1038/bjc.2016.194 (PMC4947705; doi:10.1038/bjc.2016.194)
Supplement: Supplementary Figure 1 [file bjc2016194x3.ppt]

## Slide 1
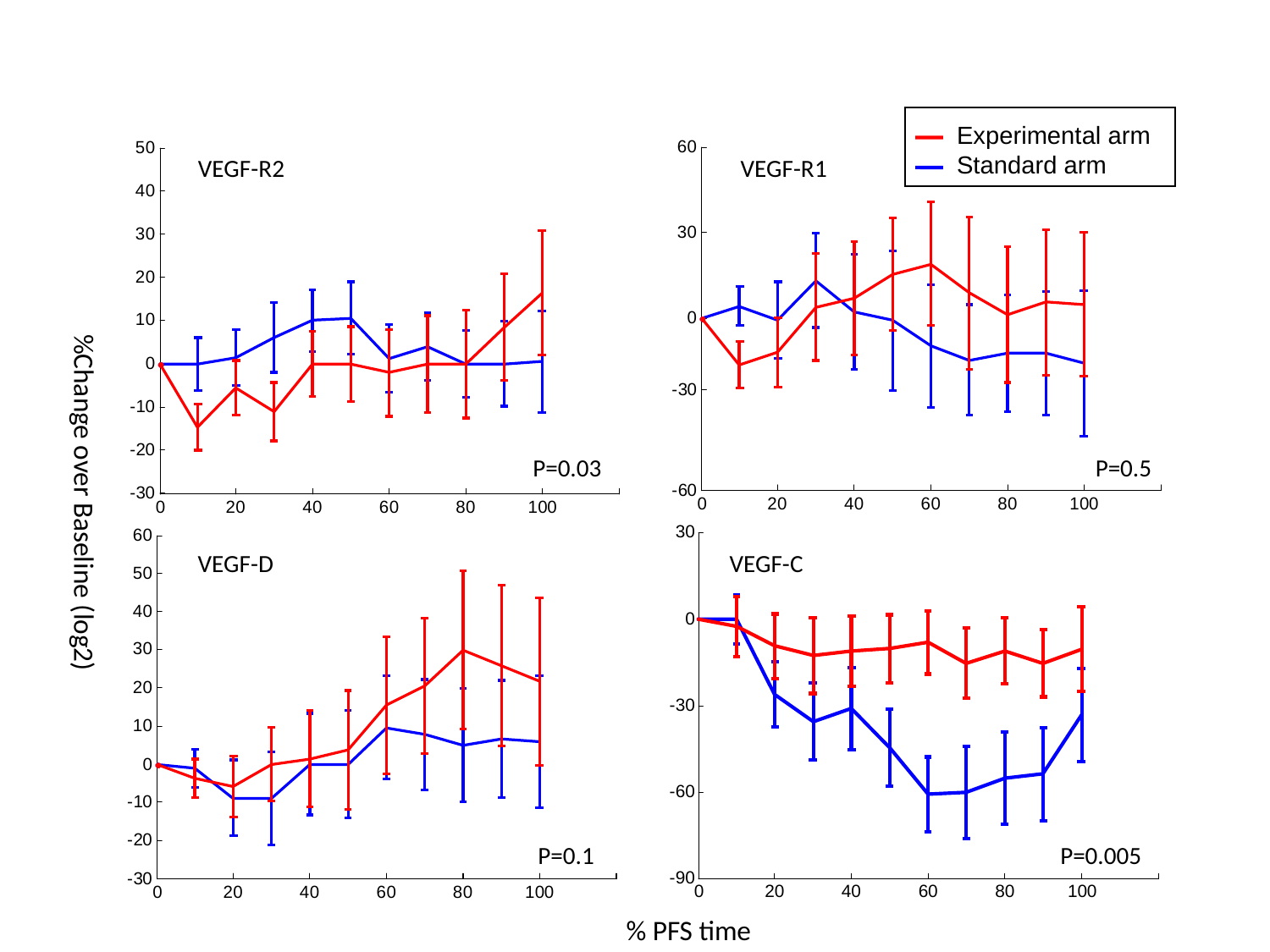

Experimental arm
Standard arm
VEGF-R2
VEGF-R1
%Change over Baseline (log2)
P=0.03
P=0.5
VEGF-D
VEGF-C
P=0.1
P=0.005
% PFS time

## Slide 2
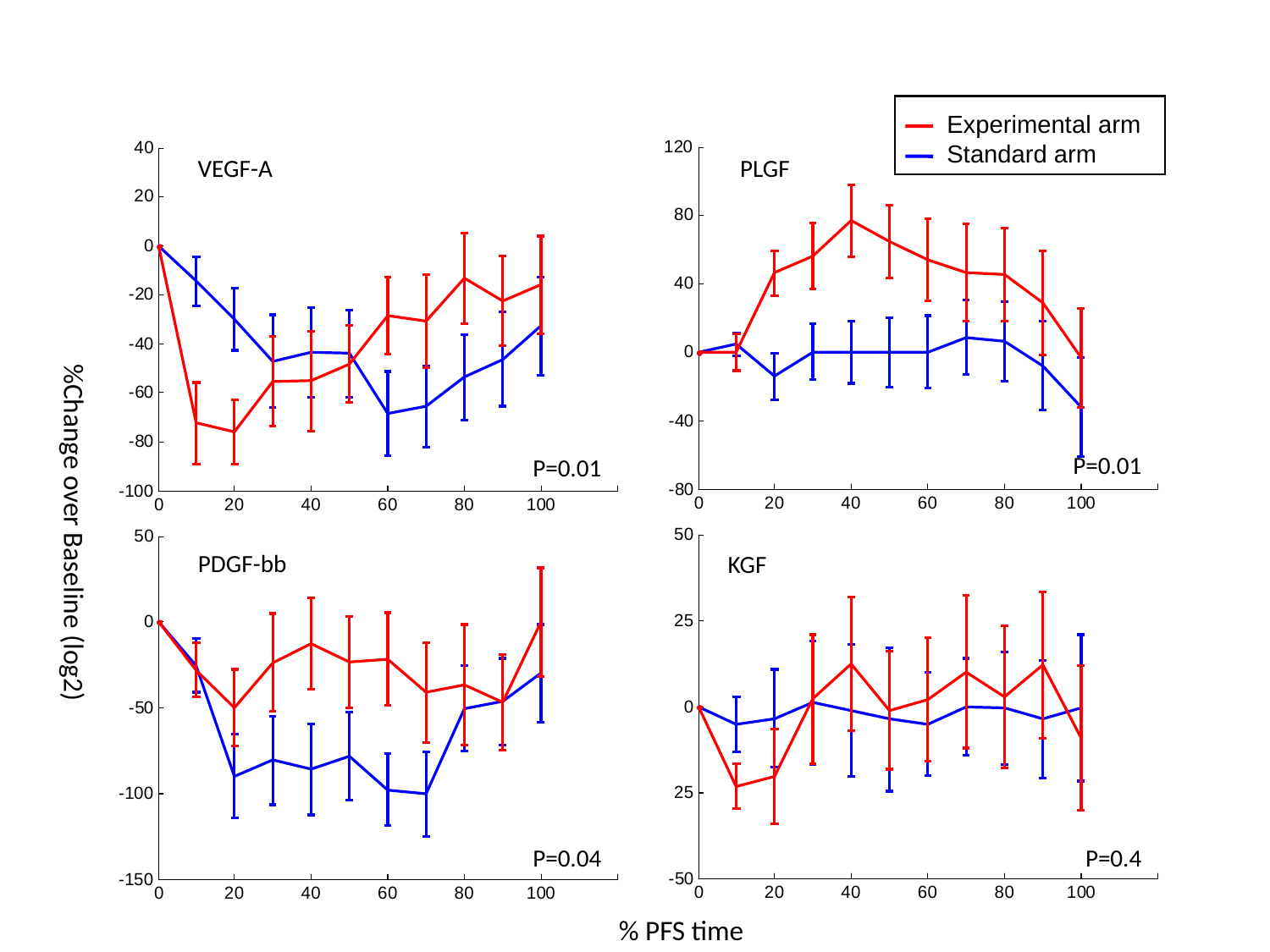

Experimental arm
Standard arm
VEGF-A
PLGF
%Change over Baseline (log2)
P=0.01
P=0.01
PDGF-bb
KGF
P=0.04
P=0.4
% PFS time

## Slide 3
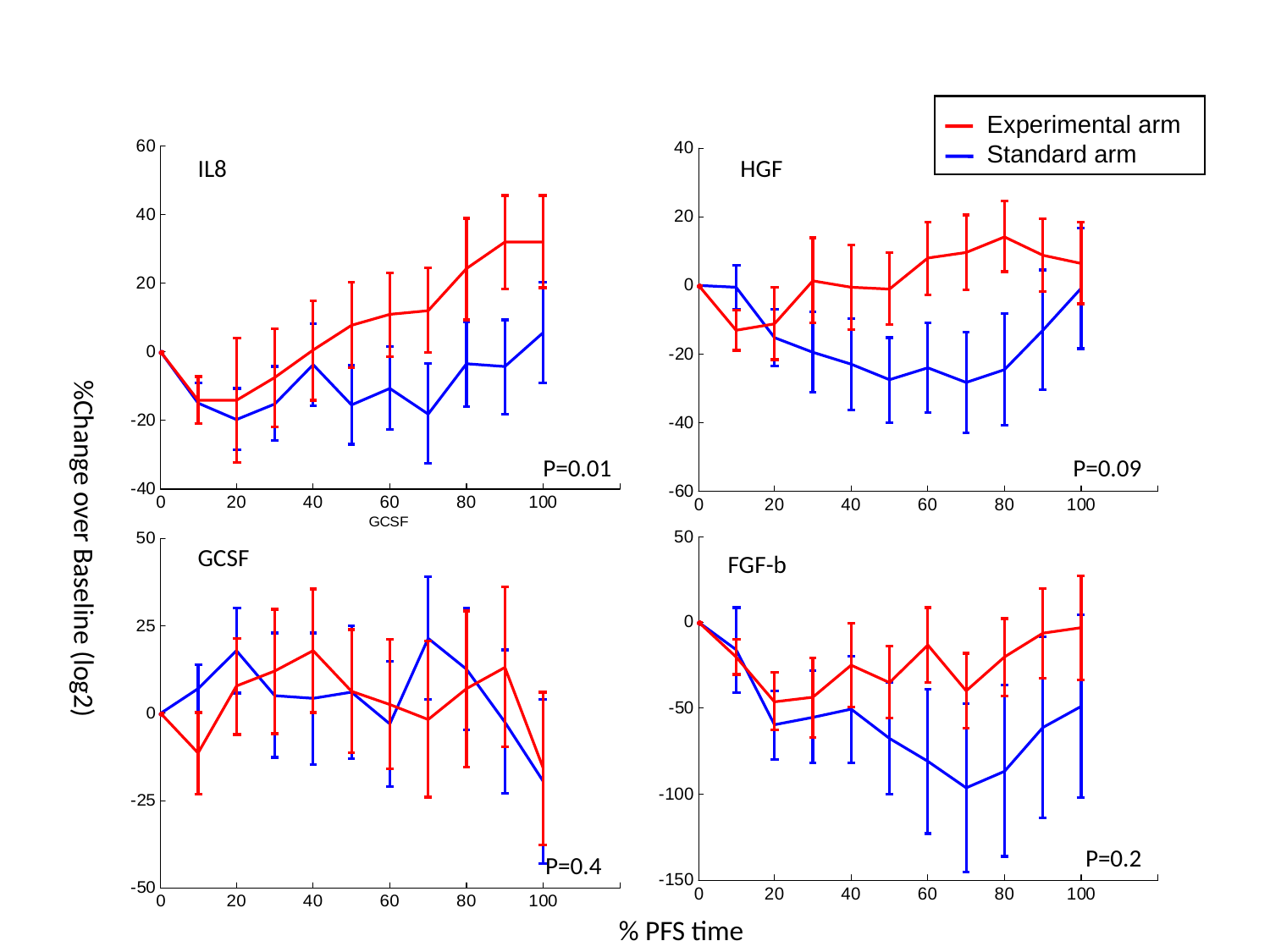

Experimental arm
Standard arm
IL8
HGF
%Change over Baseline (log2)
P=0.01
P=0.09
GCSF
FGF-b
P=0.2
P=0.4
% PFS time

## Slide 4
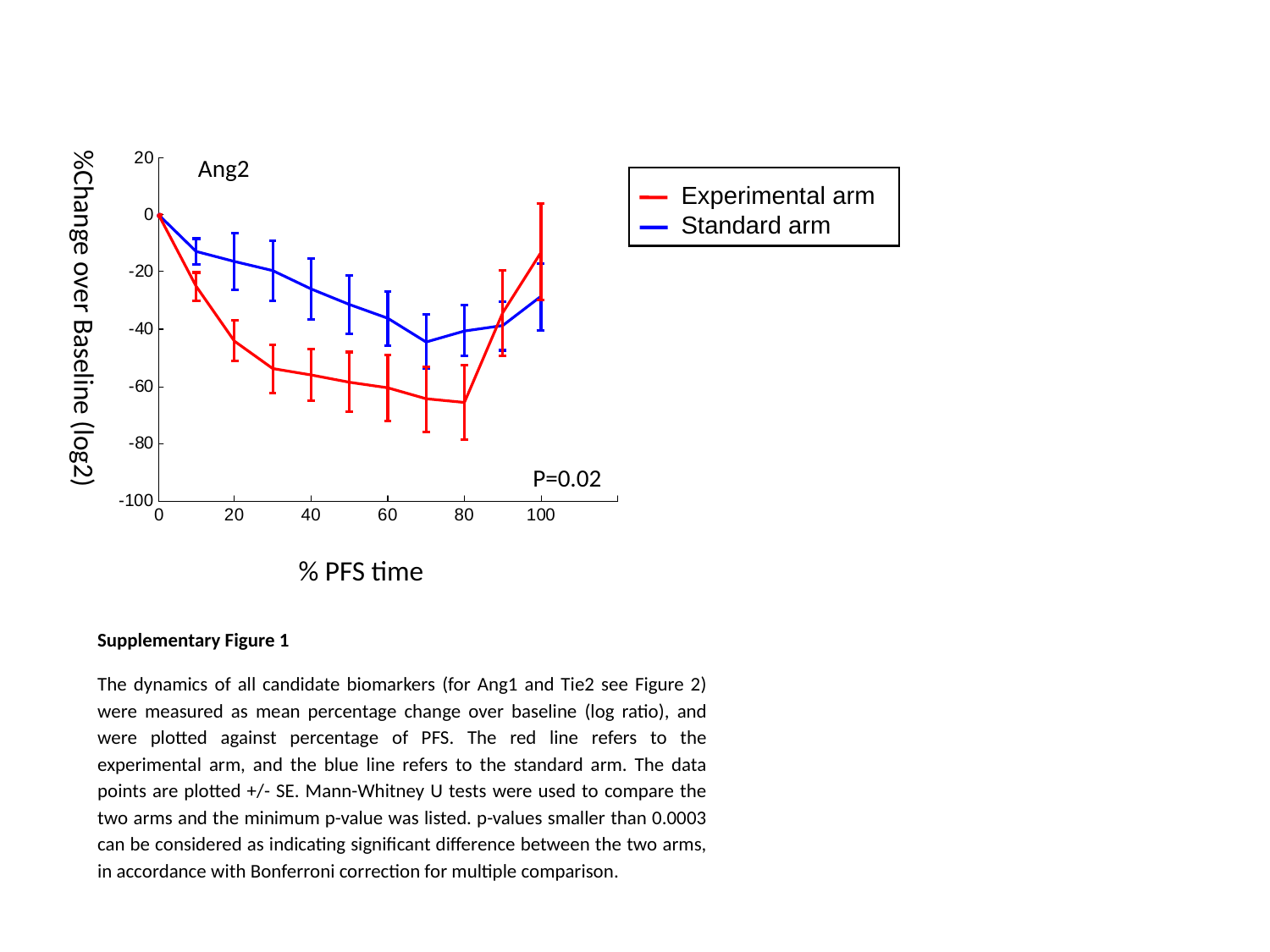

%Change over Baseline (log2)
Ang2
Experimental arm
Standard arm
P=0.02
% PFS time
Supplementary Figure 1
The dynamics of all candidate biomarkers (for Ang1 and Tie2 see Figure 2) were measured as mean percentage change over baseline (log ratio), and were plotted against percentage of PFS. The red line refers to the experimental arm, and the blue line refers to the standard arm. The data points are plotted +/- SE. Mann-Whitney U tests were used to compare the two arms and the minimum p-value was listed. p-values smaller than 0.0003 can be considered as indicating significant difference between the two arms, in accordance with Bonferroni correction for multiple comparison.
